# Increment in the volcanic unrest and number of eruptions after the 2012 large earthquakes sequence in Central America

**DOI:** 10.1038/s41598-021-01725-1

**Published:** 2021-11-17

**Authors:** Gino González, Eisuke Fujita, Bunichiro Shibazaki, Takumi Hayashida, Giovanni Chiodini, Federico Lucchi, Izumi Yokoyama, Karoly Nemeth, Raúl Mora-Amador, Aaron Moya, Gustavo Chigna, Joan Martí, Dmitri Rouwet

**Affiliations:** 1 Volcanes sin Fronteras, San José, Costa Rica; 2 International Institute of Seismology and Earthquake Engineering, Building Research Institute, Tsukuba, Japan; 3 National Graduate Institute for Policy Studies (GRIPS), Tokyo, Japan; 4 Dipartimento di Scienze della Terra e Geoambientali, Università degli studi di Bari Aldo Moro, Bari, Italy; 5 Istituto Nazionale di Geofisica e Vulcanologia, Sezione di Bologna, Bologna, Italy; 6 National Research Institute for Earth Science and Disaster Resilience, Tsukuba, Japan; 7 Department of Biological, Geological and Environmental Sciences, University of Bologna, Bologna, Italy; 8 The Japan Academy, Ueno Park, Tokyo, Japan; 9 Volcanic Risk Solutions, School of Agriculture and Environment, Massey University, Palmerston North, New Zealand; 10 Institute of Earth Physics and Space Science, Sopron, Hungary; 11 Laboratorio de Ingeniería Sísmica (LIS-UCR), Universidad de Costa Rica, San José, Costa Rica; 12 Instituto Nacional de Sismología, Vulcanología, Meteorología e Hidrología, Ciudad de Guatemala, Guatemala; 13 Geosciences Barcelona, CSIC, Barcelona, Spain

**Keywords:** Natural hazards, Solid Earth sciences

## Abstract

Understanding the relationship cause/effect between tectonic earthquakes and volcanic eruptions is a striking topic in Earth Sciences. Volcanoes erupt with variable reaction times as a consequence of the impact of seismic waves (i.e. dynamic stress) and changes in the stress field (i.e. static stress). In 2012, three large (*M*_*w*_ ≥ 7.3) subduction earthquakes struck Central America within a period of 10 weeks; subsequently, some volcanoes in the region erupted a few days after, while others took months or even years to erupt. Here, we show that these three earthquakes contributed to the increase in the number of volcanic eruptions during the 7 years that followed these seismic events. We found that only those volcanoes that were already in a critical state of unrest eventually erupted, which indicates that the earthquakes only prompted the eruptions. Therefore, we recommend the permanent monitoring of active volcanoes to reveal which are more susceptible to culminate into eruption in the aftermath of the next large-magnitude earthquake hits a region.

## Introduction

“Was the volcanic eruption triggered by the earthquake?” The answer to this question is usually “maybe” or “it could be a coincidence”. These ambiguous answers are due to the lack of observational data and/or clear scientific evidence relating these two processes. Darwin^1^, in his expedition to Chile in 1835, witnessed the Concepción earthquake in February of that year, and noted the subsequent increase in activity in some volcanoes. Based on this observation, he proposed the possibility of a relation between earthquakes and volcanic eruptions. Observed cases of volcanic eruptions supposedly caused by tectonic earthquakes have been reported in different tectonic settings. For example, the Plinian eruption of Mt. Fuji (an arc volcano situation on the Japanese subduction front), was preceded by a gigantic megathrust earthquake *M*_*w*_ = 8.7 (Hoei earthquake in 1707), 49 days before^2^. In the extension zone of Iceland occurred an increment in the volcanic eruptions after the earthquakes sequence of 1618 and 1789^3^. In the case of a hot spot volcano, the *M*_*w*_ = 7.7 Kalapana earthquake (Hawaii) in 1975 promoted rift-zone intrusions in Kilauea volcano and unleashed the 1977 eruption^4^. Nonetheless, some research has examined this question from an opposite standpoint and focusses instead on how volcanic activity increases seismicity^5,6^.

Alterations in volcanic activity are observed after an increase in seismicity at both short (< 100 km) and long (> 500 km) distances from the epicenter^7–10^. Other manifestations related to seismic events observed in volcanoes include variations in deformation rates^7,11,12^, degassing and heat flux^13–15^, and phreatic activity^16–19^.

Some studies suggest that volcanoes can react to a tectonic earthquake very quickly, in just a few hours or days^20^. These reactions are assumed to be triggered by the dynamic stress caused by the seismic waves transmitted through the volcanic system^7,10,16^.

Other studies discussed that volcanoes will tend to erupt in the medium-to-long term, that is, months, years or even decades after the earthquakes^7,9,21–23^. These longer reaction times can be explained by (1) the co-seismic pressure change in the stress field around the volcano or in the magma system, known as static stress^7–9^ and/or in the case of giant megathrust earthquakes in convergent tectonic systems by (2) the viscoelastic relaxation of the mantle and its effect on the magmatic system. An example of these long reaction times is the increase in the number of eruptions per year in the Cascades (western North America), for over a century after the *M*_*w*_ = 9.0 giant earthquake that hit the region in 1700 AD^7^. Recently, studies have suggested that giant earthquakes can create subsidence, which instigates the horizontal movement of magma bodies and hydrothermal systems; an example of such a process would be the *M*_*w*_ = 8.8 Maule (Chile) and *M*_*w*_ = 9.0 Tohoku (Japan) earthquakes in 2010 and 2011, respectively^11,12^.

Yet, it is not clear why some volcanoes change their behavior after a tectonic earthquake, although the majority of them do not. Recent research has proposed that an earthquake alone is not enough to trigger an eruption, if the magmatic system is not ready to erupt^8,24,25^.

This study investigates the unique occurrence of three major earthquakes in Central America, generated by the subduction of the Cocos plate beneath the Caribbean plate, over a period of just 72 days in 2012 (August 27, El Salvador, *M*_*w*_ = 7.3; September 5, Costa Rica, *M*_*w*_ = 7.6; November 7, Guatemala, *M*_*w*_ = 7.4) and how these earthquakes affected the state of unrest or eruption in the volcanoes of the region. Here, we conduct a statistical analysis (Monte Carlo approach^26^) and numerical modelling of the stress regime of selected volcanoes that erupted after this series of earthquakes and demonstrate that only those that were in a clear state of unrest before the arrival of the seismic waves actually ended up erupting.

### Tectonic earthquakes and volcanic activity in Central America

This study focusses on Central America, a region with all of the necessary “ingredients” for our purpose: large earthquakes and dozens of active volcanoes. The historical record of earthquakes and volcanic eruptions are since 1528 and 1524, respectively. However, this historical information is inconsistent regarding the exact location and magnitude of the earthquakes, and is incomplete in terms of the number of volcanic eruptions, which is a hindrance for any robust statistical analyses.

**Figure 1:**
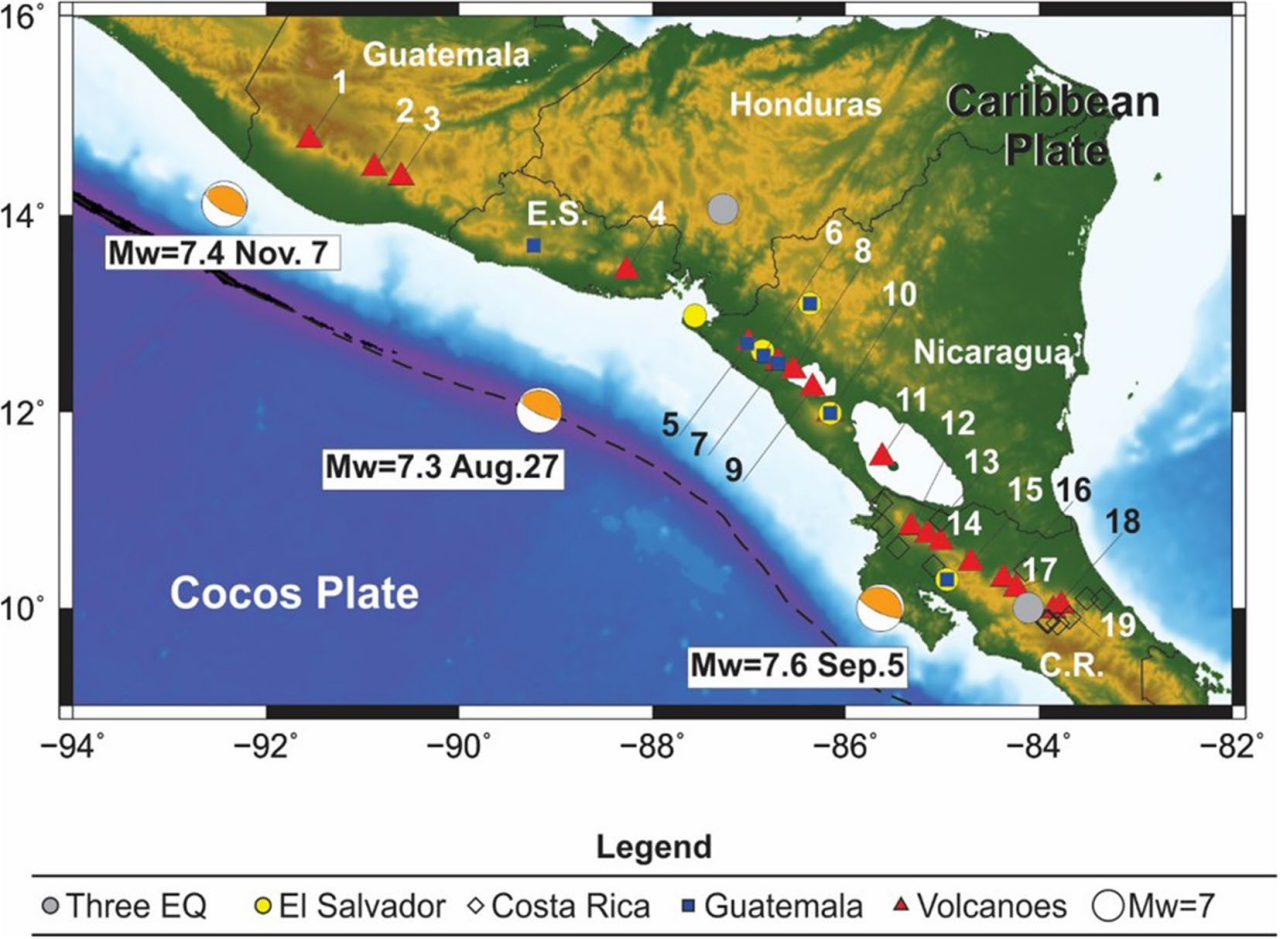
Epicenter of the 2012 earthquakes, volcanoes in states of unrest, and location of the seismic stations used to obtain the waveforms for calculating the dynamic stress of the earthquakes in Central America (more information in the Supplementary Material). The dashed line corresponds to the Meso-American trench along which the Cocos plate is subducting below the Caribbean plate. Grey circles indicate the seismic stations available for the three earthquakes in 2012 (August 27, El Salvador; September 5, Costa Rica; November 7, Guatemala). The yellow circles, black diamonds, and blue squares indicate the seismic stations that generated information for the El Salvador, Costa Rica and Guatemala earthquakes, respectively. The orange/white circles are the focal mechanism of each earthquake from Global CMT. The volcanoes analyzed in this study are: 1. Santa María, 2. Fuego, 3. Pacaya, 4. San Miguel, 5. San Cristóbal, 6. Telica, 7. Cerro Negro, 8. Momotombo, 9. Apoyeque, 10. Masaya, 11. Concepción, 12. Rincón de la Vieja, 13. Miravalles, 14. Tenorio, 15. Arenal, 16. Platanar, 17. Poás, 18. Irazú and 19. Turrialba. Figure created in Generic Mapping Tools (GMT; https://www.generic-mapping-tools.org/).

Nevertheless, for the last two decades, the available data set for the region is complete because each country (Guatemala, El Salvador, Nicaragua and Costa Rica) has its own seismic and volcano observatory agencies and offices of Civil Protection. Seismic and volcanism programs and data-bases now exist globally, and social networks help to spread the news and other information exceedingly quickly. With this in mind, our study is anchored in 2012, the year in which three large earthquakes of magnitudes *M*_*w*_ = 7.3 (August 27), *M*_*w*_ = 7.6 (September 5) and *M*_*w*_ = 7.4 (November 7) struck El Salvador, Costa Rica, and Guatemala, respectively, within a period of just 10 weeks (Fig. 1). After the first two earthquakes, some volcanoes resumed volcanic unrest (Fig. 1). In particular, San Cristóbal (Nicaragua) and Fuego (Guatemala) volcanoes had large eruptions just a few days after the two events and many people had to evacuate their homes around these volcanoes. Both eruptions generated pyroclastic density currents (PDC) that burned vegetation and killed livestock^27^. In other volcanoes that were already erupting, (e.g. Santa María and Fuego) the number of eruptions and explosivity increased after these three earthquakes. Notably, the number of paroxysmal explosions in Fuego increased drastically after the 2012 earthquakes (21 paroxysmal events in 1999–2012 and over 55 paroxysmal eruptions in 2012–2018^28,29^).

In the following years, other volcanoes increased their levels of unrest, which in some cases culminated in large volcanic eruptions (e.g., Telica, Rincón de la Vieja, Poás and Turrialba). Some volcanoes, which passed through decades of volcanic quiescence, resumed their magmatic activity after the earthquakes series: San Miguel, Momotombo, Rincón de la Vieja, Poás, and Turrialba erupted after 37, 110, 18, 62 and 148 years of volcanic quiescence, respectively.

The aim of our present research was to investigate whether or not the three large earthquakes in 2012 promoted the increase in volcanic activity in Central America in the short- and/or long-terms. Here, we considered the characteristics of the earthquakes and the pattern of activity in each one volcano prior to the series of earthquakes as key parameters for unravelling why some volcanoes erupted and others did not.

## Results

### Increase in volcanic activity after the 2012 earthquakes

**Figure 2:**
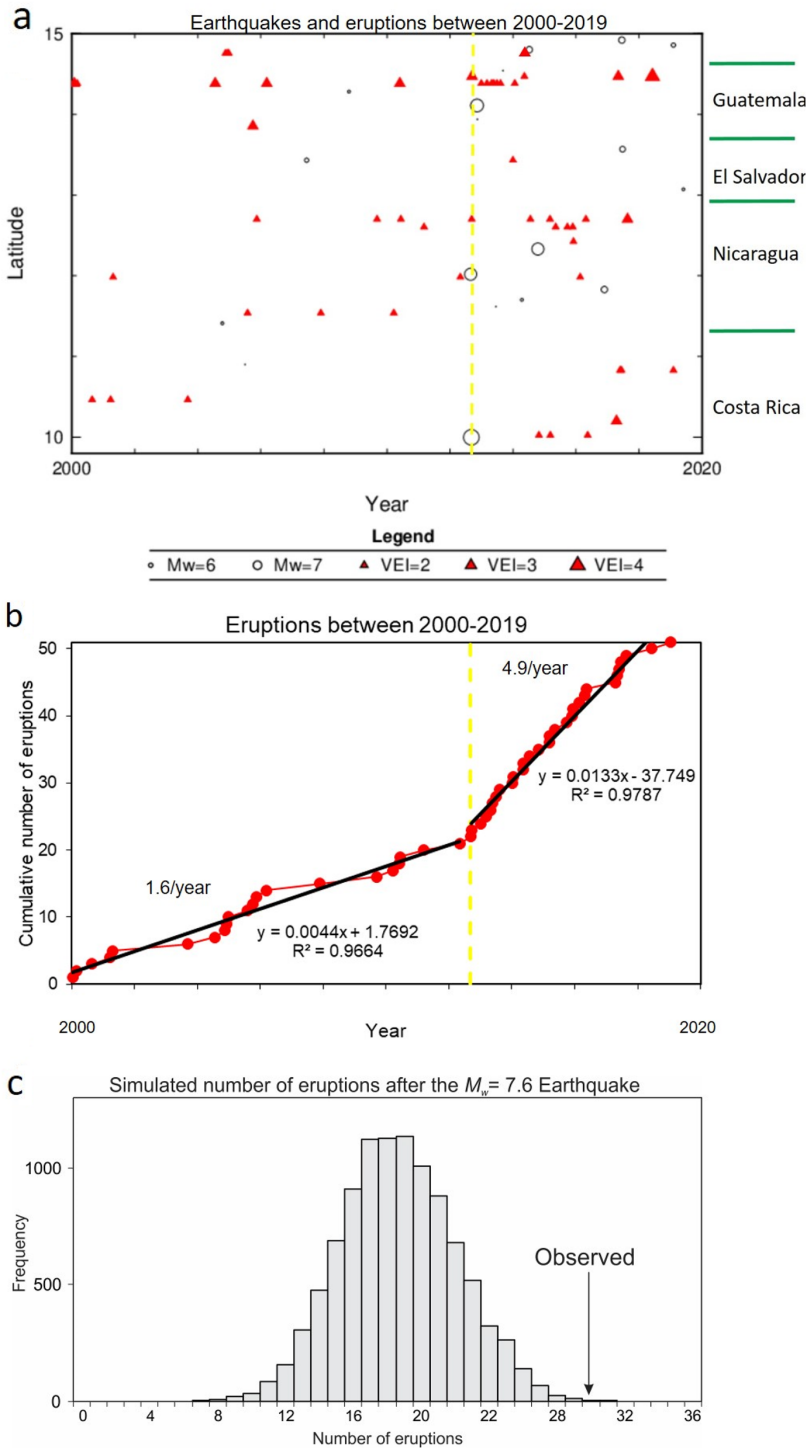
Volcanic eruptions in Central America during the period of 2000–2019 (for reference see Supplementary Material, Table S5) and the Monte Carlo simulation (see “Methodology” section). (**a**) Volcanic eruptions by size and locations in Central America; (**b**) the cumulative number of volcanic eruptions showing the increase in the eruption rate after the 2012 earthquakes; (**c**) histogram of the number of eruptions calculated from 10,000 random simulations after the Costa Rica earthquake using the Monte Carlo simulation and the observed data. The yellow dashed lines in (**a,b**) correspond to the Costa Rica earthquake on September 5, 2012.

In the period of 2000–2019, 51 volcanic eruptions with a Volcanic Explosive Index^30^ (VEI) ≥ 2 occurred in Central America, of which 21 were from before the three large 2012 earthquakes and 30 were afterwards (Fig. 2a; see Supplementary Material, Table S5). This observation corresponds to an increase in the annual eruption rate from 1.6 to 4.9 before and after the 2012 earthquakes, respectively (Fig. 2b). From a visual qualitative comparison, the observed change in the cumulative eruption rate is unlikely to be the product of a random process. We hence applied a Monte Carlo simulation^26^ to discriminate whether or not this increase in the number of volcanic eruptions could have been a random process or linked to a cause/effect relationship (see “Methodology” section). We ran 10,000 random simulations and only 12 of them (0.12%) provided results that were similar to the observed data (Fig. 2c). Thus, the testing hypothesis can be rejected using standard confidence levels (e.g. 0.01), thereby suggesting that the observed acceleration in the number of volcanic eruptions was not a simple coincidence, and in fact reflects a significant change induced by an external factor: the earthquakes occurred exactly at the point at which the curve of the cumulative number of volcanic eruptions changes its slope.

### Stress changes caused by the earthquakes

The San Cristóbal and Fuego volcanoes erupted 3 and 8 days after the Costa Rica earthquake, respectively. This quick reaction could have been caused by a disturbance in the system prompted by the dynamic stress. We calculated the dynamic stress (σ_D_) using the seismic waveform and the static stress differential (σ_sdiff_), maximum (σ_smax_) and minimum (σ_smin_) in each analyzed volcano in response to the three large earthquakes (see Supplementary Material, Tables S1, S2, Fig. S1). San Cristóbal volcano was subject to σ_D_ = 0.022 MPa and 0.255 MPa and Fuego volcano σ_D_ = 0.013 MPa and 0.031 MPa by El Salvador and Costa Rica earthquakes, respectively. In the case of the static stress, only the El Salvador earthquake produced more than 1 kPa on San Cristóbal volcano, which underwent a change of σ_sdiff_ = 2.5 kPa, σ_smax_ = 1.5 kPa and σ_smin_ = − 1.2 kPa in its N-S alignments. The static stress produced by the Costa Rica earthquake was less than 1 kPa for both volcanoes, this a magnitude that represents a negligible change in the stress regime.

After the August 27 El Salvador earthquake, San Miguel volcano received the maximum dynamic stress (0.16 MPa). This volcano also underwent the most significant change in its static stress with a σ_sdiff_ = 3 kPa, σ_smax_ = 2 kPa and σ_smin_ =  − 2 kPa given an alignment of 160°. For the September 5, Costa Rica earthquake, Rincón de la Vieja volcano was subjected to 1.25 MPa of dynamic stress. Furthermore, this volcano experienced the largest change in the static stress regime as a result of this earthquake with a σ_diff_ = 55 kPa, σ_smax_ = 40 kPa σ_smin_ =  − 15 kPa, with W–E alignment. Santa María volcano, on the other hand, underwent a maximum σ_D_ = 0.39 MPa generated by the November 7 Guatemala earthquake. This earthquake caused the largest change in static stress, with a σ_sdiff_ = 0.1 MPa, σ_smax_ = 80 kPa and σ_smin_ =  − 30 kPa, in an alignment of 60°.

## Discussion and conclusions

### Volcanic eruptions in 2000–2019

As demonstrated in Fig. 2a–c, after the 2012 earthquakes, the number of volcanic eruptions effectively increased along the Central American Volcanic Arc (hereafter, CAVA). In some volcanoes the number of eruptions and explosivity increased compared to previous years (Santa María and Fuego), while others such as San Miguel, Momotombo, Rincón de la Vieja, Poás and Turrialba volcanoes began to erupt. The eruption rate increased by a factor of 3.0. A similar trend was observed after the *M*_*w*_ = 9.3 Andaman-Sumatra earthquake (December 24, 2004), where there was a fourfold increase in the eruption rate in the region, due mainly to the expansion in the volcanic system^22^. However, after the 2012 Central America earthquakes, the volcanic eruptions occurred diachronously: some volcanic eruptions occurred shortly afterwards (within a matter of days), and other months or even years after the earthquakes. Nevertheless, no migration of the volcanic eruption based on the location and time was obvious, except for two volcanic eruptions clustered temporally (weeks to months after the earthquakes) and spatially close to each other. This is the case for the volcanic eruptions series of Fuego and Pacaya volcanoes and San Cristóbal, Telica and Momotombo volcanoes (Fig. 2a). The latter sequence, occurred after the *M*_*w*_ = 6.1, April 10, 2014 and *M*_*w*_ = 7.3, October 14, 2014 earthquakes.

### Earthquakes characteristics

**Figure 3:**
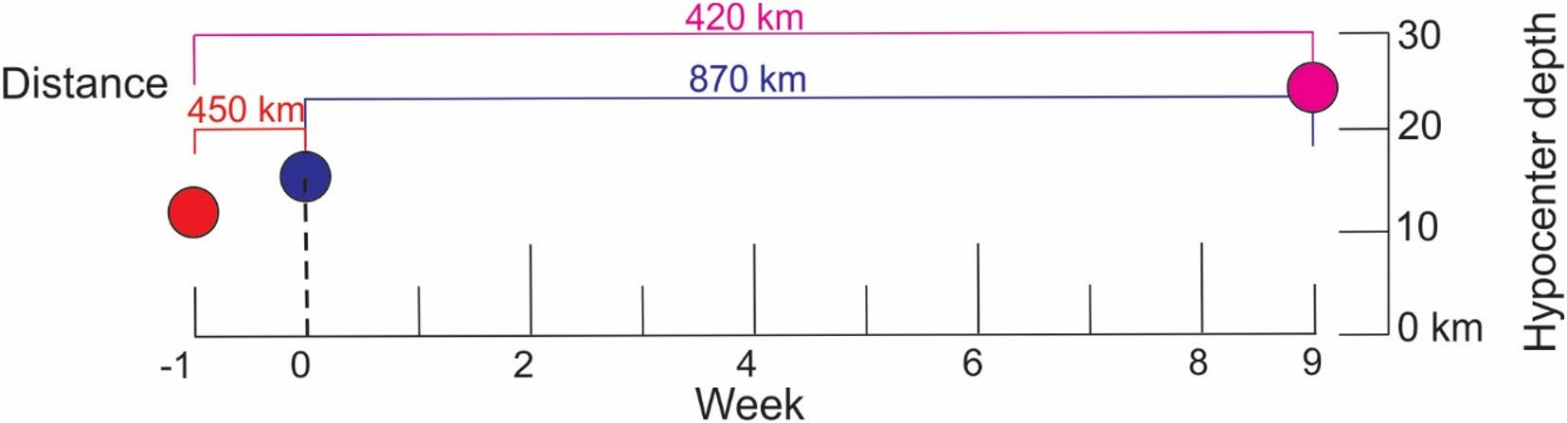
Distance, hypocenter depth and time occurrence of the 2012 earthquakes in El Salvador (red), Costa Rica (blue) and Guatemala (cyan).

The three large earthquakes occurred within 10 weeks of each other, almost equidistantly (420–450 km), but at different hypocenter depths (Fig. 3). The hypocenter depth of the El Salvador earthquake (August 27, week 1, *M*_*w*_ = 7.3) was 11.8 km and had low high-frequency (HF) energy radiation and a long period, which are typical characteristics of “tsunamigenic earthquakes”^31–35^. Nine days later, the Costa Rica earthquake (September 5, week 0, *M*_*w*_ = 7.6) struck with moderate HF energy radiation and with a hypocenter depth of 15.8 km^32,34,35^. The hypocenter depth of the Guatemala earthquake (November 7, week 9, *M*_*w*_ = 7.4) was 24 km, where conditionally stable areas surround small patches in the slab that, at failure, produced a moderate slip and high HF radiation^32,34,35^.

The spectra of the El Salvador and Costa Rica earthquakes ranged from 0.07 to 1.2 Hz, with a frequency domain of 0.07–0.1 Hz in broadband stations. It is important to consider both, the resonance frequency of the fluids^36^ (i.e. magma, gas, vapour, and liquid) as well of the volcanic edifice^37^, in order to evaluate whether or not Fuego and San Cristóbal volcanoes could have entered into resonance and squeezed out magma after crack opening. We calculated the resonance frequency of theoretical magma-filled conduits, i.e., dykes (*f*_*rd*_) with hypothetical widths of 100 m and 10 m to be 0.09 and 0.28 Hz, respectively. The resonance frequency of the volcanic edifices calculated (*f*_*rv*_) for Fuego volcano is around 0.16 Hz and for San Cristóbal volcano is around 0.27 Hz (for more details of these calculations, see the Supplementary Material). These resonance frequencies of the fluid-filled and volcanic edifices of Fuego and San Cristóbal volcanoes are within or close to the range of the frequency domain of the earthquakes, which means, that it is possible that this mechanism prompted these eruptions.

Dynamic stress is generally considered for time intervals of just a few seconds. However, in the case of the El Salvador earthquake, Telica volcano experienced stress lasting around 120 s (as opposed to the typical 20–50 s^14^), at the same continuous frequency as well as the corresponding, continuous dynamic stress. This could have caused the sloshing mechanism in the hydrothermal and/or magmatic plumbing system^36,38^.

In addition, sloshing depends on viscosity^36,38^, silicic magmas being more viscous than mafic magmas. For San Cristóbal and Fuego volcanoes, the predominant magmas in recent eruptions are basaltic-andesitic, which means that the overpressure needed to trigger an eruption is lower than for dacitic/rhyolitic magmas.

### Volcanic eruptions shortly after the earthquakes

On September 8 and 13, there was a paroxysmal eruption with VEI = 2 and VEI = 3 at San Cristóbal and Fuego volcanoes, respectively. To evaluate whether or not these eruptions were possibly triggered by the earthquakes, we calculated a lithostatic pressure in the magma reservoir^24^ of 98 MPa (magma chamber depth: 4000 m of San Cristóbal volcano) and 73.5 MPa (magma chamber depth: 3000 m of Fuego volcano), respectively (see Supplementary Material). The total change in the lithostatic pressure produced by σ_D_ of the Costa Rica earthquake was 0.26% for San Cristóbal and 0.02% for Fuego. This estimate implies that the earthquake itself could not have triggered the eruptions. Nevertheless, the disturbance in the stress regime created by the earthquake could have favoured other mechanisms—including rectified diffusion, bubble growing, an increase in the dissolved gas or magma migration, etc^7,16,25,39^—that may in turn have facilitated the eruptions after the earthquake. In addition, as explained above, it is likely that these volcanoes entered in resonance and experienced sloshing.

Regarding the role of the static stress, the respective alignment system could be crucial^40,41^. In the case of San Cristóbal, the σ_sdiff_ = 2.5 kPa, was located around the zone of high and low rigidity, i.e. the contact between the country-rock and magma chamber boundary (depth 3000 m) (more details in the Methodology and Supplementary Material, Tables S3, S4). Hence, σ_sdiff_ is a potentially good parameter for predicting crack opening and could have induced crack propagation and consequent fluid migration^8^. Unlike in San Cristóbal, the static stress of Fuego volcano was less than 1 kPa, which is arguably too low to provoke crack opening and fluid migration. Nevertheless, some studies suggest that a change in stress of just a few kPa can trigger volcanic eruptions, as has occurred on Etna and Stromboli (both in Italy)^42,43^, and Merapi (Indonesia)^44^, three of the world’s most frequently erupting volcanoes. Geological evidence from the 2006 Merapi eruption, shows that on the preceding May 26, 2006 the Yogyakarta earthquake (*M*_*w*_ = 6.4) added xenoliths from the carbonate basement to the magma chamber, thereby causing an internal pressure increase generated by CO_2_, which eventually culminated in an eruption^45^.

### Volcanic eruptions long after the earthquakes

For the August 27 El Salvador earthquake, the static stress applied was low, in the order of ± 2 kPa in three volcanoes (San Miguel, San Cristóbal and Telica). In the case of the September 5, Costa Rica earthquake, the σ_sdiff_ was from 5 kPa for Turrialba to 55 kPa for Rincón de la Vieja. The σ_sdiff_ in the November 7 Guatemala earthquake ranged from 12 kPa for Pacaya to 0.1 MPa for Santa María. The static stress in Karymsky volcano (in Kamchatka, Russia) produced by a tectonic earthquake (*M*_*w*_ = 7.1) that led to dyke intrusion and triggered the 1996 eruption was 0.2 MPa^46^, which means that it is difficult to explain with our results alone how static stress could have opened cracks and generated a dyke intrusion. Quantifying the stress regime around each volcano is a necessary constraint for determining whether the static stress reduces or increases the country-rock strength. An example of how the static stress changes according to the alignment is provided by Rincón de la Vieja volcano; where the stress regime had at least three directions (N–S, W–E and 45°). The largest σ_sdiff_ (55 kPa) occurred in the deepest part of the magma chamber with a W–E alignment. This differential pressure could cause magma to rise towards the shallow reservoir, thereby leading to overpressure and the superheating of the shallow magma chamber. Another factor that could be related to the crack propagation and/or dyke intrusion is the increase in the temperature which reduces the frictional coefficient along the fractures^41^. After the 2012 earthquakes, the temperature of fumaroles and/or acid lakes on Telica, Rincón de la Vieja, Poás and Turrialba volcanoes, increased; these volcanoes erupted years later, which indicates how magmatic intrusion can occur over an extended period of time.

The possible responses of volcanoes in the long term (months to years) to earthquakes depend on the degree of the critical stage of each volcano, which explains why some of the studied volcanoes that received more stress (dynamic and static) than others responded later, or not at all. For example, Rincón de la Vieja and Poás volcanoes erupted in 2017 and underwent more stress change than Turrialba volcano, which had already erupted in 2014. Volcanic processes such as magma migration from the mantle to the crust or magma mixing, can occur on various time scales, from months to years or even centuries^47,48^. In addition, the presence of a mush zone, part of which could be an eruptible melt at crustal depth, a seismic event or some other process, such as the addition of a mafic melt, can trigger eruptions years later (e.g. the deadly phreatic eruption of Ontake volcano in 2014^49^).

Another hypothesis regarding the increase in the number of eruptions even years after the 2012 earthquakes, is the post-seismic activity in Central America. The region is well known for the occurrence of post-seismic slow-slip earthquakes, as was the case of the El Salvador and Costa Rica 2012 earthquakes^50,51^. A good example is provided by San Miguel volcano, which erupted in December 2013, after 37 years of quiescence. According to GPS data, the horizontal displacement by the co-seismic slip at San Miguel volcano was around 1.2 cm^50^. Nevertheless, almost one year after the three earthquakes, the horizontal displacement reached 2 cm due to the post-seismic slip^50^.

Although some of the 19 studied volcanoes had already erupted prior to the 2012 earthquakes, the number of eruptions increased after the earthquakes. For Poás volcano, the number and magnitude of phreatic explosions increased after the January 8, 2009 Cinchona earthquake^17–19^, a *M*_*w*_ = 6.2 tectonic event with an epicenter 10 km from Poás and also after the 2012 earthquakes. On April 10, 2014, a *M*_*w*_ = 6.1 tectonic earthquake hit near Momotombo volcano (Nicaragua), triggered seismic swarms^52^ and resumed explosive activity in December 2015. We cannot rule out the possibility that other strong earthquakes such as the 2014 Nicaragua earthquakes or even the Chiapas earthquake (*M*_*w*_ = 8.2, 7 September, 2017) have affected the volcanic system. Indeed, the “cocktail” of earthquakes could provide extra stress (dynamic and static) in volcanoes that were at the point of erupting. The most impressive change in the increase in the number of eruptions took place on Fuego volcano: in just three years (2015–2018), 50 paroxysmal eruptions occurred, including the deadly eruption of June 3, 2018, which is far more than the 21 paroxysmal eruptions that occurred in 1999–2012^29^ (n.b. the Fuego eruption is still ongoing, but the most recent data are not included in the present study).

### Volcanic unrest for the period of 2007–2012

**Figure 4:**
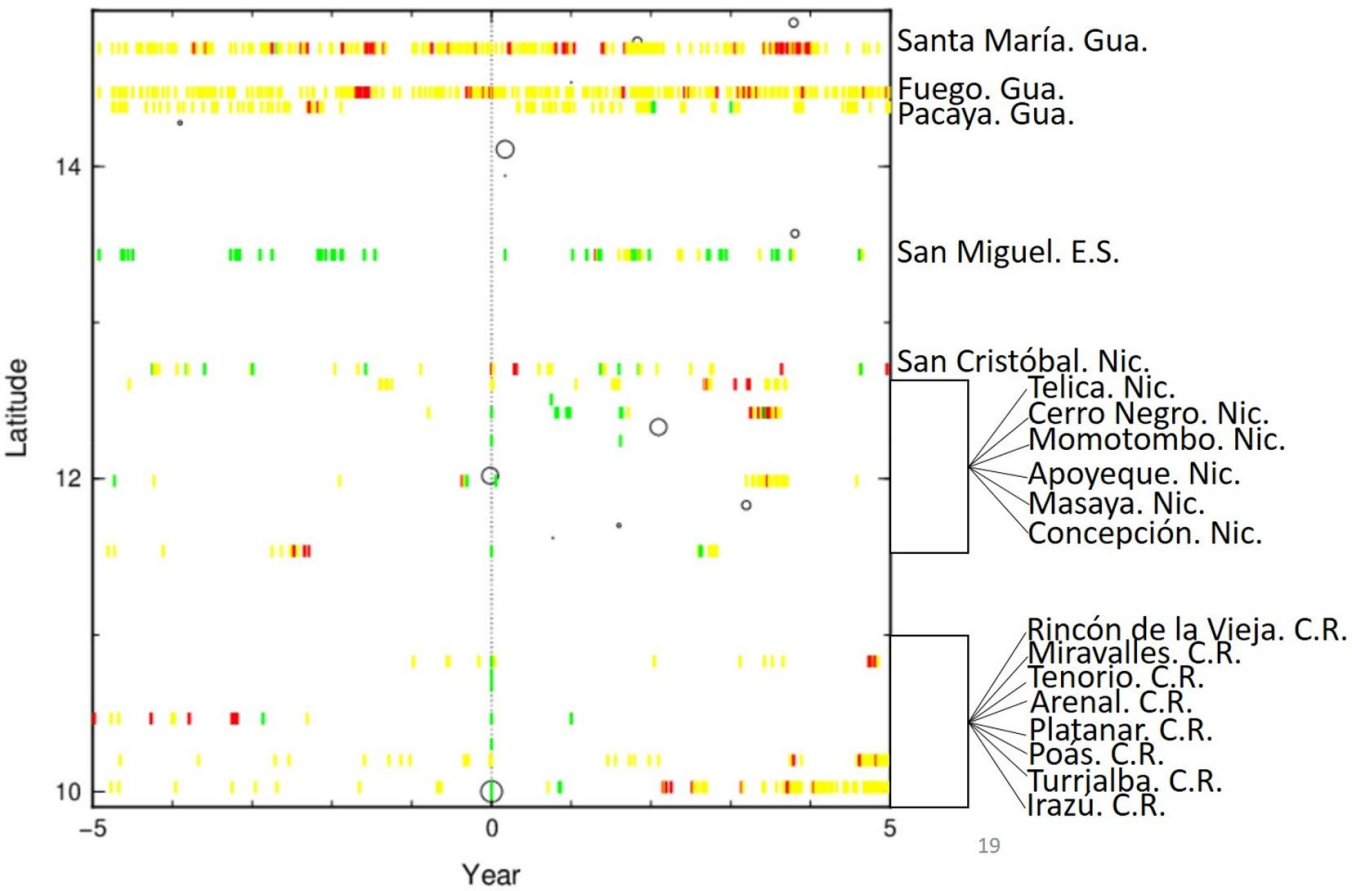
Volcanic unrest and earthquakes from 2007 (− 5) to 2017 (5). Dotted line (0) corresponds to the Costa Rica earthquake on September 5, 2012. Green represents the increase in seismicity in the volcanic system. Yellow indicates an increase in the temperature, deformation, degassing or, phreatic activity, or a number of small explosions. Red is related to the occurrence of large eruptions with considerable ashfall, explosions with ballistics and paroxysmal events. Blank space indicates that no information is available or that the volcanoes were in a state of quiescence.

The change in volcanic activity from background behavior to worrisome levels (i.e. volcanic unrest) sometimes escalates into volcanic eruptions or triggers other hazard events^53–56^. Our research categorized the information available on volcanic activity into three different “degrees of unrest”, determined by the energy released of volcanoes^57^ and running in a range from the lowest degree (unrest 1), through intermediate degree (unrest 2), to the highest degree (unrest 3) degree. These degrees of unrest can be described thus: *Unrest 1* = increase in the seismicity of the volcanic system (green in Fig. 4); *Unrest 2* = increase in the temperature, deformation, degassing, and phreatic activity, or the occurrence of small explosions (yellow in Fig. 4); *Unrest 3* = occurrence of large eruptions with considerable ashfall, explosions with ballistics and paroxysmal events (red in Fig. 4). Between September 2007 and September 2017, 19 volcanoes in the CAVA showed signs of unrest before and/or after the earthquakes of 2012 (Figs. 1, 4). Before the 2012 earthquakes, 13 volcanoes were in a state of unrest (Santa María, Pacaya, Fuego, San Miguel, San Cristóbal, Telica, Momotombo, Masaya, Concepción, Rincón de la Vieja, Arenal, Poás and Turrialba; Fig. 4), and, of these, Santa María, Fuego, Pacaya, San Cristóbal, Telica, Masaya, Concepción, and Arenal were erupting. After the earthquakes and in the years up to 2017, Concepción, and Arenal ceased to erupt. A phreatomagmatic event occurred on Concepción volcano in May 2011^27,58^; on Arenal volcano the magnitudes of the explosions declined constantly from 2007 onwards^27^, and the last explosion occurred in October 2010. After the 2012 earthquakes, these volcanoes decreased their level of unrest to 1 or 2 (Fig. 4). A possible reason why these volcanoes may not have erupted after the 2012 earthquakes could lie on the fact that the magma volume erupted previously had already lowered the internal pressure in the close-conduit system of these two volcanoes. The other volcanoes in eruption prior to September 2012—Santa María, Fuego, Pacaya, San Cristóbal, Telica and Masaya—are all very active open-conduit systems and/or are in permanent unrest, and so their internal pressure constantly reaches the threshold to triggering eruptions.

Despite decades without any magmatic eruptions, some of the other volcanoes already had unrest degrees of 1 or 2 (San Miguel, Momotombo, Rincón de la Vieja, Poás and Turrialba; Figs. 1, 4) prior to the 2012 earthquakes. Of the 19 volcanoes in a state of unrest in the period 2007–2017, eleven volcanoes erupted after the 2012 earthquakes, all of which were already in a state of unrest before the 2012 earthquakes. This begs the question: why did the other eight volcanoes not erupt? A number of answers are possible (Fig. 4): (1) two of them had already released their energy during large eruptions and/or prolonged periods of activity, as suggested above (Concepción and Arenal); (2) five volcanoes showing no previous signs of unrest switched into unrest only after the Costa Rica earthquake occurred (Apoyeque, Miravalles, Tenorio, Platanar and Irazú; Fig. 4). Of these five volcanoes, only one volcano had ever erupted in historical times (Irazú, 1963–1965), while the other four volcanoes are far from the recurrence period for a potential new eruption. These five volcanoes only reached unrest degree 1 (increased seismicity) some hours or a few days after the Costa Rica earthquake. This response can be linked to the dynamic stress that triggered a number of seismic swarms in the fault systems around these volcanoes^8,10,59^. (3) large eruption occurred on Cerro Negro in August 1999 and unrest degree 1 was reached on June 4, 2013. The behavior from these eight volcanoes indicate that the earthquakes themselves were insufficient to trigger volcanic eruptions, despite the fact that the earthquakes caused an increase in the degree of unrest in some cases. However, the same seismic energy transmitted to the other volcanoes that were already in an advanced state of unrest was sufficient to trigger a new eruption. In consequence, we postulate that, in view of the data presented and the obtained results, the dormant volcanoes or volcanoes with low activity levels did not change their states to any significant degree simply as a result of the effects of the shaking generated by the earthquake, or the change in the stress field regime. The earthquakes were not able by themselves to bring the volcanoes from equilibrium to eruption.

Our findings stem from the coincidental occurrence of three subduction tectonic earthquakes in a time span of 10 weeks in the active Central American volcanic arc and lead us to conclude that the postulated cause/effect relationship between tectonic earthquakes and volcanic eruptions is only valid when volcanoes are already in a high state of unrest prior to an earthquake. The energy supplied by the seismic shock may constitute the additional energy contribution necessary to trigger an eruption in a high stage of pre-eruption volcanic activity. The fact that the volcano may react shortly, or long term after the seismic input, does not seem to depend on the magnitude of the earthquake itself but, rather, on the processes that occur inside the volcano (type of magma, gas content, viscosity, strength of the hots rock, etc.). Other earthquake characteristics in addition to magnitude and location (i.e., energy radiated, frequency, duration, etc.) may also play a role in explaining how tectonic earthquakes contribute to volcanic eruptions. Nevertheless, this external energy supply, regardless of the distance between the earthquake epicenter and the volcano and the magnitude of the event, is not sufficient to raise the state of a volcano from quiescence directly into eruption. Our results confirm the need to monitor all active volcanoes as a means of establishing their degree of unrest at any particular time and hence prior to the next large earthquake, as well as the need to determine future scenarios for possible increased volcanic activity and eventually for volcanic eruption in the short (days) or long (years) terms. This kind of surveillance is a key forecasting tool for future eruptions, and will help Civil Protection authorities and other decision-makers to adopt appropriate strategies to disaster risk reduction at the regional or local scales.

## Methodology

### Statistical analysis

The question discussed in this paper stems from the fact that there is that it seems to have been an acceleration in the number of eruptions after the large earthquakes of 2012 (Fig. 2b); the debate revolves around whether this distribution can be casual or differs significantly from a random distribution of the eruptions over time. To answer this question we investigated a random distribution by using Monte Carlo simulations^26^ (Fig. 2c). We computed the probability that a random distribution has this point with a value below the observed one, i.e., if at the time of the earthquake the number of volcanic eruptions could be lower than the observed 21 value (testing hypothesis). The simulation considered that: (a) the number of eruptions in the period of 2000–2019 = a total of 51 volcanic eruptions occurred in 7305 days (20 years); (b) the fact that the second of the three earthquakes, the *M*_*w*_ = 7.6 Costa Rica earthquake struck on September 5, 2012 (day 4632) when 21 out of 51 volcanic eruptions had already occurred; and (c) 30 out of 51 eruptions occurred after the second earthquake (i.e., between day 4632 and 7305). We ran 10,000 simulations, following the law of large number and found that only 0.12% of these simulations satisfies the testing hypothesis. Our results show that it is likely that the observed acceleration in the number of volcanic eruptions is not due to chance, but represents, instead, a significant change induced by the earthquake.

### Dynamic stress

The pressure change inside a geological system due to the passing of seismic waves is called dynamic stress (σ_D_). This can be calculated using the Eq. (1)^60^:

1 $${\sigma }_{D}=\frac{PGV\times G}{Vph},$$

where *PGV* is the peak ground velocity of the seismic wave (km/s), *G* is the shear modulus with a value of 30 GPa for the region^32^ and *Vph* is the velocity phase of the wave (km/s). The dynamic stress considers the maximum peak-to-peak velocity of the waveform (see Supplementary Material, Tables S1, S2, Fig. S1).

### Static stress

The static stress is the change in the local stress field occurring after the earthquake. The software used to calculate the static stress was “Advance FrontSTR”, which is based on a finite element method^8,61^. We created simulations of each magma chamber of spherical shape and a diameter of 1000 m with a rigidity of 1 kPa. The depth location and alignment are based on available publications; more details are in Supplementary Material, Tables S3 and S4.

The calculation of static stress is governed by the Eqs. (2) and (3):

2 $$\frac{\partial {\sigma }_{ij}}{\partial {x}_{j}}+{f}_{i}=0,$$

3 $${\sigma }_{ij}=D{\varepsilon }_{ij},$$

where σ_ij_ is the stress tensor, *f*_*i*_ is the external force vector applied, *D* is the matrix of elastic constants, and ε is the strain.

### Volcanic eruptions for the period of 2000–2019

We recognized volcanic eruptions occurring in the Central American region with a Volcanic Explosive Index^30^ (VEI) ≥ 2, and reported with its day, month, and year of occurrence from January 1, 2000 to December 31, 2019. In the case of phreatic explosions, the lack of volcanic deposits and the existence of confusing reports are typical and so we only took into account the eruptions with a well-defined VEI, despite the occurrence of phreatic eruptions at some volcanoes in the region (e.g. Poás, Turrialba) during the study period.

### Volcanic unrest between 2007 and 2017

This research delimited a period of 5 years before and 5 years after the earthquakes^9^ for determining whether or not the earthquakes that occurred in 2012 increased volcanic unrest. The catalog compiled for the states of volcanic unrest of the 19 volcanoes included information from internal reports by local observatories, scientific papers, and personal data in addition to the weekly report from the Global Volcanism Program (GVP)^27^.

## Supplementary Information

Supplementary Information.

## Data Availability

The authors declare that the data supporting the findings of this study are available within the supplementary information files.
