# Author Correction: Increment in the volcanic unrest and number of eruptions after the 2012 large earthquakes sequence in Central America

**DOI:** 10.1038/s41598-021-03918-0

**Published:** 2021-12-23

**Authors:** Gino González, Eisuke Fujita, Bunichiro Shibazaki, Takumi Hayashida, Giovanni Chiodini, Federico Lucchi, Izumi Yokoyama, Karoly Nemeth, Raúl Mora-Amador, Aaron Moya, Gustavo Chigna, Joan Martí, Dmitri Rouwet

**Affiliations:** 1 Volcanes sin Fronteras, San José, Costa Rica; 2 International Institute of Seismology and Earthquake Engineering, Building Research Institute, Tsukuba, Japan; 3 National Graduate Institute for Policy Studies (GRIPS), Tokyo, Japan; 4 Dipartimento di Scienze della Terra e Geoambientali, Università degli studi di Bari Aldo Moro, Bari, Italy; 5 Istituto Nazionale di Geofisica e Vulcanologia, Sezione di Bologna, Bologna, Italy; 6 National Research Institute for Earth Science and Disaster Resilience, Tsukuba, Japan; 7 Department of Biological, Geological and Environmental Sciences, University of Bologna, Bologna, Italy; 8 The Japan Academy, Ueno Park, Tokyo, Japan; 9 Volcanic Risk Solutions, School of Agriculture and Environment, Massey University, Palmerston North, New Zealand; 10 Institute of Earth Physics and Space Science, Sopron, Hungary; 11 Laboratorio de Ingeniería Sísmica (LIS-UCR), Universidad de Costa Rica, San José, Costa Rica; 12 Instituto Nacional de Sismología, Vulcanología, Meteorología e Hidrología, Ciudad de Guatemala, Guatemala; 13 Geosciences Barcelona, CSIC, Barcelona, Spain

Correction to: *Scientific Reports* https://doi.org/10.1038/s41598-021-01725-1, published online 17 November 2021

The original version of this Article contained a repeated error in the Introduction, in Figure 1 and its accompanying legend, in the Results section under the subheading ‘Stress changes caused by the earthquakes’, in the Discussion and conclusions section under the subheading ‘Volcanic eruptions long after the earthquakes’, and in the Supplementary Information file, where the earthquake that occurred on November 7, 2012 was incorrectly mentioned as having occurred on November 11, 2012. The original Fig. 1 and accompanying legend appear below.

**Figure 1:**
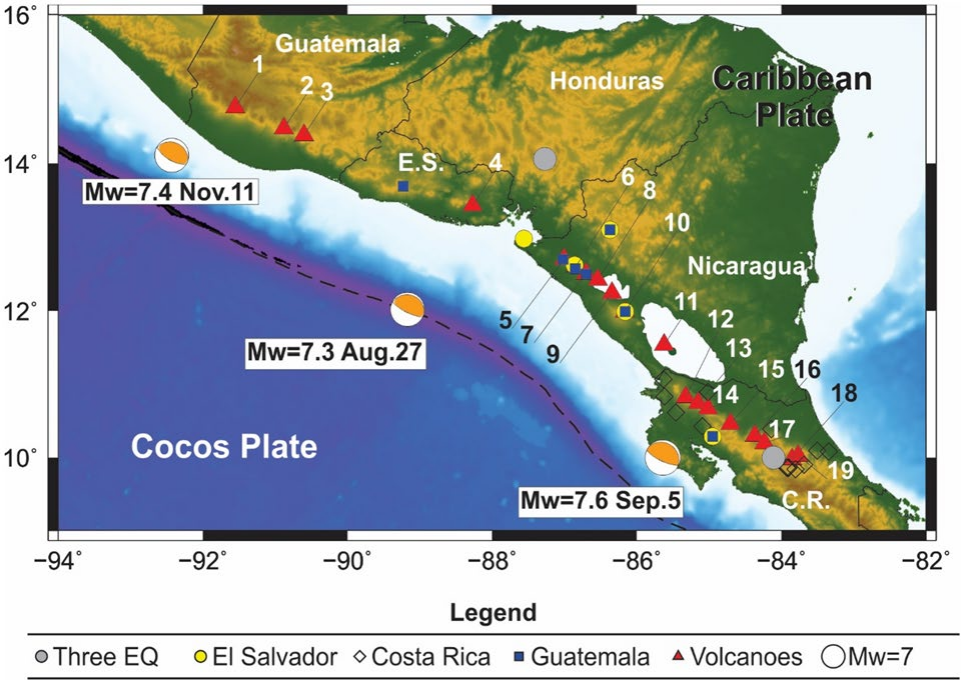
Epicenter of the 2012 earthquakes, volcanoes in states of unrest, and location of the seismic stations used to obtain the waveforms for calculating the dynamic stress of the earthquakes in Central America (more information in the Supplementary Material). The dashed line corresponds to the Meso-American trench along which the Cocos plate is subducting below the Caribbean plate. Grey circles indicate the seismic stations available for the three earthquakes in 2012 (August 27, El Salvador; September 5, Costa Rica; November 11, Guatemala). The yellow circles, black diamonds, and blue squares indicate the seismic stations that generated information for the El Salvador, Costa Rica and Guatemala earthquakes, respectively. The orange/white circles are the focal mechanism of each earthquake from Global CMT. The volcanoes analyzed in this study are: 1. Santa María, 2. Fuego, 3. Pacaya, 4. San Miguel, 5. San Cristóbal, 6. Telica, 7. Cerro Negro, 8. Momotombo, 9. Apoyeque, 10. Masaya, 11. Concepción, 12. Rincón de la Vieja, 13. Miravalles, 14. Tenorio, 15. Arenal, 16. Platanar, 17. Poás, 18. Irazú and 19. Turrialba. Figure created in Generic Mapping Tools (GMT; https://www.generic-mapping-tools.org/).

The original Article and the Supplementary Information file that accompanies the original Article have been corrected.

