# Supplementary material for: Increment in the volcanic unrest and number of eruptions after the 2012 large earthquakes sequence in Central America
Source: Sci Rep. 2021 Nov 17;11:22417. doi: 10.1038/s41598-021-01725-1 (PMC8599426; doi:10.1038/s41598-021-01725-1)
Supplement: Supplementary file 1 — Supplementary Information. [file 41598_2021_1725_MOESM1_ESM.docx]

**Title:**

INCREMENT IN THE VOLCANIC UNREST AND NUMBER ERUPTIONS AFTER THE 2012 LARGE EARTHQUAKES SEQUENCE IN CENTRAL AMERICA

Gino González 1,2,3,4,5*, Eisuke Fujita 6, Bunichiro Shibazaki 2, Takumi Hayashida 2, Giovanni Chiodini 5, Federico Lucchi 7, Izumi Yokoyama 8, Karoly Nemeth 9,10, Raúl Mora-Amador 11, Aaron Moya 12, Gustavo Chigna 13, Joan Martí 14, Dmitri Rouwet 5.

1 Volcanes sin Fronteras, Costa Rica.

2 International Institute of Seismology and Earthquake Engineering, Building Research Institute, Tsukuba, Japan.

3 National Graduate Institute for Policy Studies (GRIPS), Tokyo, Japan.

4 Dipartimento di Scienze della Terra e Geoambientali, Università degli studi di Bari Aldo Moro, Bari, Italy.

5 Istituto Nazionale di Geofisica e Vulcanologia, Sezione di Bologna, Bologna, Italy.

6 National Research Institute for Earth Science and Disaster Resilience, Tsukuba, Japan.

7 Department of Biological, Geological and Environmental Sciences, University of Bologna, Bologna, Italy.

8 The Japan Academy, Ueno Park, Tokyo, Japan.

9 Volcanic Risk Solutions, School of Agriculture and Environment, Massey University, New Zealand.

10 Institute of Earth Physics and Space Sciences, Sopron, Hungary.

11 Private Consultant in Geology, San José, Costa Rica.

12 Laboratorio de Ingeniería Sísmica (LIS-UCR), Universidad de Costa Rica, San José, Costa Rica.

13 Instituto Nacional de Sismología, Vulcanología, Meteorología e Hidrología, Guatemala.

14 Geosciences Barcelona, CSIC, Spain.

*Corresponding author [ginovolcanico@gmail.com](mailto:ginovolcanico@gmail.com)

**Supplementary material**

This research considered (1) three large tectonic earthquakes that occurred in 2012 in Central America, (2) the volcanic unrest five years before and five years after these earthquakes, and (3) volcanic eruptions with a Volcanic Explosive Index^1^ (VEI) ≥2 from 2000 to 2019.

*Seismic waveforms of the 2012 earthquakes*

The waveform of each earthquake is necessary to calculate the dynamic stress. In the case of the August 27, 2012 El Salvador earthquake, the first of the three, seven waveforms are available from IRIS (Supplementary Table 1). For the September 5, 2012 Costa Rica earthquake, two waveforms from IRIS and 16 accelerometer data from LIS-UCR are available (Supplementary Table 1). In the case of the November 7, 2012 Guatemala earthquake, nine waveforms are available from IRIS. We use the broadband seismic signals of IRIS. To obtain the dynamic stress we applied the equation^2^ (1):

$$\sigma_{D}=\frac{PGV*G}{Vph}$$

(1)

where *PGV* is the peak ground velocity of the seismic wave (km/s), *G* is shear modulus with a value of 30 GPa for the region^3^ and *Vph* is the velocity phase of the wave (km/s). The dynamic stress considers the maximum peak-to-peak velocity of the waveform.

**Supplementary Table 1.** Dynamic stress_,_ calculated in each seismic station analyzed in Central America induced by the large earthquakes of 2012. E.S. EQ is El Salvador earthquake; CR EQ correspond with Costa Rica earthquake and GUA EQ means Guatemala earthquake. Distance to EQ is the distance in kilometer of the seismic station to the earthquake epicenter. Peak to Peak refers to the peak ground velocity of the seismic waveform (cm/s). σ_D_ abs is the absolute dynamic stress in MPa.

| Station | Latitude | Longitude | Earthquake | Distance to EQ (km) | Peak to Peak (m/s) | σ_D_ abs (MPa) |
| --- | --- | --- | --- | --- | --- | --- |
| CSGN | 12.98 | -87.56 | E.S. EQ | 1.51E+02 | 3.61E-03 | 0.028 |
| TBHS | 12.62 | -86.86 | E.S. EQ | 2.03E+02 | -5.94E-03 | 0.052 |
| MASN | 11.99 | -86.16 | E.S. EQ | 2.88E+02 | 1.41E-03 | 0.012 |
| JTS | 10.29 | -84.95 | E.S. EQ | 4.53E+02 | 8.25E-04 | 0.007 |
| HDC | 10.00 | -84.11 | E.S. EQ | 5.50E+02 | -5.55E-04 | 0.005 |
| TGUH | 14.06 | -87.27 | E.S. EQ | 2.61E+02 | 5.27E-03 | 0.046 |
| ESTN | 13.10 | -86.37 | E.S. EQ | 2.71E+02 | 1.73E-03 | 0.015 |
| HDC | 10.00 | -84.11 | CR EQ | 1.40E+02 | 5.95E-02 | 0.467 |
| TGUH | 14.06 | -87.27 | CR EQ | 4.91E+02 | 1.73E-02 | 0.150 |
| AALA | 10.03 | -84.21 | CR EQ | 1.56E+02 | 8.96E+00 | 0.703 |
| AFRA | 10.14 | -84.19 | CR EQ | 1.59E+02 | 8.11E+00 | 0.637 |
| AUPA | 10.90 | -85.01 | CR EQ | 1.21E+02 | -8.06E+00 | 0.632 |
| CCHI | 9.84 | -83.80 | CR EQ | 2.02E+02 | -5.66E+00 | 0.444 |
| CCRT | 9.86 | -83.93 | CR EQ | 1.88E+02 | -6.46E+00 | 0.507 |
| CSRH | 9.87 | -83.90 | CR EQ | 1.91E+02 | -5.31E+00 | 0.416 |
| CTEC | 9.85 | -83.91 | CR EQ | 1.90E+02 | 3.79E+00 | 0.297 |
| CTUH | 9.90 | -83.69 | CR EQ | 2.14E+02 | 2.87E+00 | 0.225 |
| GCNS | 10.43 | -85.09 | CR EQ | 7.66E+01 | -1.31E+01 | 1.024 |
| GJTS | 10.28 | -84.96 | CR EQ | 8.04E+01 | -1.74E+01 | 1.366 |
| GLCR | 11.07 | -85.63 | CR EQ | 1.19E+02 | -1.34E+01 | 1.053 |
| GLIB | 10.62 | -85.46 | CR EQ | 7.15E+01 | -1.59E+01 | 1.250 |
| GSTR | 10.84 | -85.62 | CR EQ | 9.31E+01 | -1.36E+01 | 1.068 |
| HVRG | 10.39 | -84.14 | CR EQ | 1.70E+02 | 4.67E+00 | 0.366 |
| LBTN | 10.08 | -83.35 | CR EQ | 2.51E+02 | 6.80E+00 | 0.534 |
| LSQR | 10.10 | -83.50 | CR EQ | 2.34E+02 | 5.09E+00 | 0.400 |
| TBHS | 12.57 | -86.85 | GUA EQ | 5.86E+02 | 5.18E-03 | 0.045 |
| CNGN | 12.50 | -86.70 | GUA EQ | 6.06E+02 | 1.70E-03 | 0.013 |
| MASN | 11.99 | -86.16 | GUA EQ | 6.94E+02 | -2.13E-03 | 0.017 |
| JTS | 10.29 | -84.95 | GUA EQ | 8.78E+02 | 1.05E-03 | 0.008 |
| SNET | 13.69 | -89.23 | GUA EQ | 3.09E+02 | 5.80E-03 | 0.051 |
| CRIN | 12.70 | -87.02 | GUA EQ | 5.66E+02 | -4.12E-03 | 0.032 |
| HDC | 10.00 | -84.11 | GUA EQ | 9.74E+02 | -1.16E-03 | 0.010 |
| TGUH | 14.06 | -87.27 | GUA EQ | 5.18E+02 | -2.03E-03 | 0.018 |
| ESTN | 13.10 | -86.37 | GUA EQ | 6.25E+02 | 2.54E-03 | 0.022 |

This research choses seismic stations principally located around the volcanoes. However, not all volcanoes are covered with seismic stations, and in these cases, some stations located close to the volcano can help to understand how the dynamic stress changes regionally. All the dynamic stress showed a tendency to decay with the distance^4–6^. However, as shown in Supplementary Figure 1, every earthquake had its own decay, due to the earthquake characteristic such as frequency and magnitude and frequency recorded in the instrument. Some stations showed a site effect problem such as for AUPA and LBTN (the Costa Rica earthquake), while large attenuation occurred as CNGN. In addition, some stations did not have the three components (CNGN, HDC); in these cases, to reduce the error in the interpolation, we only used the vertical component.


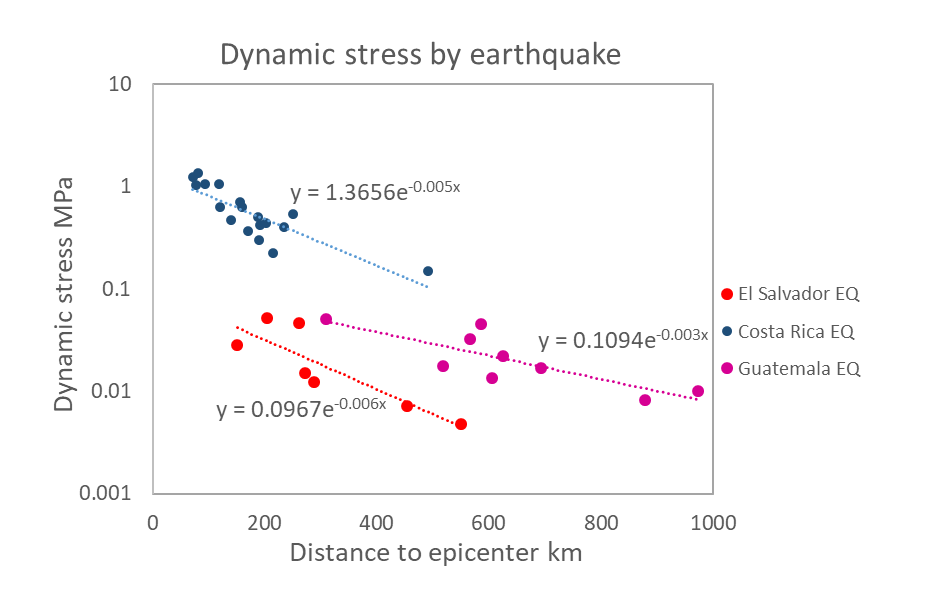


**Supplementary Figure 1.** Dynamic stress in MPa calculated in the logarithmic scale and the distance from the epicenter in km for each earthquakes of Central America in 2012.

In the case of the El Salvador and Guatemala earthquakes, some volcanoes are less than 200 km from the epicenter and no waveform was available. In addition, the lack of seismic stations installed in the volcanoes with unrest was a problem. To solve this, in the first case, this research considered *PGV* from USGS, and used a V_s_= 3.8 km/s to calculate the dynamic stress for San Miguel volcano in the case of the El Salvador earthquake and, Santa María, Fuego and Pacaya volcanoes for the Guatemala earthquake. Secondly, an equation can be deduced to calculate the dynamic stress for each earthquake from Supplementary Figure 1. These equations depend on the distance from the epicenter to the volcano. Equations (2-4) show these for the El Salvador, Costa Rica and Guatemala earthquakes, respectively:

$$\sigma_{D}=0.0967*e^{-0.006x}$$

(2)

$$\sigma_{D}=1.3656*e^{-0.005x}$$

(3)

$$\sigma_{D}=0.1094*e^{-0.003x}$$

(4)

where *x* is the distance from the epicenter to the volcano (in km). This method is used to simulate the dynamic stress. However, these equations are applied for volcanoes without seismic stations and only consider the absolute value in the stress. Some volcanoes have seismic stations on the summit, or within a distance of less than 25 km. In these cases, we used the real values of the dynamic stress measurements. Supplementary Table 2 shows the dynamic stress for each volcano, depending on each earthquake.

**Supplementary Table 2.** Dynamic stress (σ_D_) in MPa_,_ calculated in each volcano in Central America induced by the large earthquakes of 2012 in Central America. The red and black colors are negative and positive σ_D_, respectively. The σ_D_ are obtained using the equations 1-4.

| Volcano | σ_D_ El Salvador EQ | σ_D_ Costa Rica EQ | σ_D_ Guatemala EQ |
| --- | --- | --- | --- |
| Santa María | 8.84E-03* | 2.13E-02* | 3.92E-01** |
| Fuego | 1.34E-02* | 3.12E-02* | 1.57E-01** |
| Pacaya | 1.55E-02* | 3.61E-02* | 1.57E-01** |
| San Miguel | 1.57E-01** | 1.26E-01* | 2.79E-02* |
| San Cristóbal | 2.19E-02* | 2.55E-01* | -3.23E-02*** |
| Telica | -5.18E-02*** | 2.79E-01* | 4.51E-02*** |
| Cerro Negro | 1.87E-02* | 3.02E-01* | 1.33E-02* |
| Momotombo | 1.70E-02* | 3.26E-01* | 1.49E-02* |
| Apoyeque | 1.52E-02* | 3.71E-01* | 1.38E-02* |
| Masaya | 1.36E-02* | 4.37E-01* | -1.67E-02*** |
| Concepción | 9.32E-03* | 5.81E-01* | 1.02E-02* |
| Rincón de la Vieja | 6.92E-03* | -1.25E+00*** | 8.46E-03* |
| Miravalles | 6.11E-03* | 8.33E-01* | 7.95E-03* |
| Tenorio | 5.52E-03* | 8.23E-01* | 7.55E-03* |
| Arenal | 4.35E-03* | 7.69E-01* | 6.68E-03* |
| Platanar | 3.40E-03* | 6.67E-01* | 5.90E-03* |
| Poás | 3.06E-03* | 6.37E-01*** | 5.59E-03* |
| Irazú | 2.30E-03* | -4.16E-01*** | 4.84E-03* |
| Turrialba | 2.21E-03* | 2.25E-01*** | 4.75E-03* |

*Simulated

**USGS

***Waveform

*Fault parameter of the 2012 earthquakes*

To calculate the static stress it is necessary to set the fault parameters of each earthquake (Supplementary Table 3), the magma chamber location and the alignment in each volcano to analyze the differential, maximum and minimum static stress induced by the earthquake in different zones of the respective volcano (Supplementary Table 4).

**Supplementary Table 3.** Fault parameters of the 2012 earthquakes in Central America. The Guatemala earthquake parameter is based on the USGS finite model, N.C. = not calculated.

| Parameters | El Salvador EQ^3,7^ | Costa Rica EQ^3,7^ | Guatemala EQ |
| --- | --- | --- | --- |
| Latitude | 12.02 | 10 | 14.11 |
| Longitude | -89.17 | -85.64 | -92.43 |
| Fault 1 (m^2^) | 1.07E+09 | 8.63E+08 | 1.23E+09 |
| Fault 2 (m^2^) | 1.08E+09 | 2.02E+09 | N.C. |
| Strike (°) | 296 | 310 | 293 |
| Dip (°) | 16 | 15.7 | 28 |
| Rake (°) | 101 | 97 | 80 |
| Slip (m) | 2 | 3.9 | 4 |
| Rigidity (Pa) | 3.00E+10 | 3.00E+10 | 3.00E+10 |
| *M_o_* (N*m) | 1.29E+20 | 3.37E+20 | 1.48E+20 |
| *M_w_* | 7.3 | 7.6 | 7.4 |

**Supplementary Table 4.** Magma chamber depth and alignment in each volcano analyzed. N.D. = not data available.

| Volcano | Magma chamber depth (km) | Alignment |
| --- | --- | --- |
| Santa María | 6^8^ | 60°^9^ |
| Fuego | 3, 10^10^ | 45°* |
| Pacaya | 1, 4^11^ | 165°^11^ |
| San Miguel | 3^12^ | 160°^13^ |
| San Cristóbal | 4,16^14^ | N-S* |
| Telica | 2,4-5, 7^14^ | 30°* |
| Cerro Negro | 2, 7-8^15^ | N-S^16^ |
| Momotombo | 8^17^ | 130°* |
| Apoyeque | N.D. | 45°^18^ |
| Masaya | 3^17^ | 15°^19^ |
| Concepción | 2^20^ | 30°^21^ |
| Rincón de la Vieja | 3, 8^22^ | W-E, N-S, 45°^23^* |
| Miravalles | 3, 8^22^ | 45°^23^ |
| Tenorio | 3, 8^22^ | 160°* |
| Arenal | 3, 6^22^ | W-E* |
| Platanar | 3, 6^22^ | N-S* |
| Poás | 3^22^ | N-S* |
| Irazú | 3, 10^24^ | 150°* |
| Turrialba | 3, 10^22^ | 45°* |

* This study

*Resonance frequency*

If we propose a scenario with a vertical magma chamber (dyke) with a height (h) >> width (w), it is possible to calculate the resonance frequency (*f_rd_*) of the fluid, in our case, the magma chamber using equation (5)^25^:

$$f_{rd}=\frac{1}{2\pi}\sqrt{\frac{\pi g}{w}}$$

(5)

where *g* is the gravitational acceleration (9.8 m/s^2^). If we calculate dykes of h = 1 km and w = 100 m or 10 m, the *f_rd_* is 0.09 and 0.28 Hz, respectively. In the case of Iwate, Japan, the volcano was subjected to a frequency of 0.07 Hz for the Tohoku earthquake 2011, resulting in subsidence as evidence of the resonance^26^.

A volcanic edifice can also enter in resonance after a large earthquake. We can calculate this resonance (*f_rv_*) using equation (6)^27^ for a mountain composed of rocks only (without fluids) with h/w ratio < 0.5:

$$f_{rv}=0.7*\frac{V_{s}}{w}$$

(6)

In the case of Fuego volcano h/w = 0.15 and San Cristóbal volcano h/w = 0.14. If we consider a V_s_ = 3.8 km/s and a w = 17 km for Fuego, and w = 10 km for San Cristóbal, *f_rv_* is 0.16 and 0.27 Hz, respectively.

*Lithostatic pressure*

We calculate the lithostatic pressure (p_lit_) of the magma reservoir using equation (7)^28^:

$$p_{lit}=\rho gd$$

(7)

where ρ is rock density, assumed to be 2500 kg/m^3^, and *d* the depth of the magma chamber (m). The depth of the magma chambers are at 3000 and 4000 m for Fuego and San Cristóbal volcanoes, respectively^10,14^. The *p_lit_* in the magma chamber is 73.5 MPa for Fuego, and 98 MPa for San Cristóbal. The total change in pressure by dynamic stress was lower than 0.02% and 0.26% for Fuego and San Cristóbal, respectively, compared to the *p_lit_*.

*Volcanic eruptions between 2000-2019*

In a period of 20 years (January 1, 2000 to December 31, 2019) 50 volcanic eruptions with a VEI≥2 occurred in the Central American Volcanic Arc (CAVA hereafter, Supplementary Table 5).

Supplementary Table 5. Volcanic eruptions with a VEI≥2 in the CAVA between 2000-2019, with the corresponding date of occurrence.

| Volcano | VEI | Eruption date |
| --- | --- | --- |
| Pacaya | 3 | 14/1/2000^29^ |
| Pacaya | 2 | 29/2/2000^29^ |
| Arenal | 2 | 23/8/2000^29^ |
| Arenal | 2 | 24/3/2001^29^ |
| Masaya | 2 | 23/4/2001^29^ |
| Arenal | 2 | 5/9/2003^29^ |
| Pacaya | 3 | 19/7/2004^29^ |
| Santa María | 2 | 14/11/2004^29^ |
| Santa María | 2 | 4/12/2004^29^ |
| Santa María | 2 | 22/12/2004^29^ |
| Concepción | 2 | 28/7/2005^29^ |
| Santa Ana | 3 | 1/10/2005^30^ |
| San Cristóbal | 2 | 13/11/2005^29^ |
| Pacaya | 3 | 9/3/2006^29^ |
| Concepción | 2 | 24/11/2007^29^ |
| San Cristóbal | 2 | 6/9/2009^29^ |
| Concepción | 2 | 20/3/2010^20^ |
| Pacaya | 3 | 27/5/2010^29^ |
| San Cristóbal | 2 | 9/6/2010^29^ |
| Telica | 2 | 7/3/2011^29^ |
| Masaya | 2 | 30/4/2012^29^ |
| San Cristóbal | 2 | 8/9/2012^29^ |
| Fuego | 3 | 13/9/2012^29^ |
| Pacaya | 2 | 28/12/2012^29^ |
| Pacaya | 2 | 5/3/2013^29^ |
| Pacaya | 2 | 29/4/2013^29^ |
| Pacaya | 2 | 20/5/2013^29^ |
| Pacaya | 2 | 27/6/2013^29^ |
| Pacaya | 2 | 9/8/2013^29^ |
| San Miguel | 2 | 29/12/2013^12^ |
| Pacaya | 2 | 11/1/2014^29^ |
| Santa María | 3 | 09/5/2014^31^ |
| Fuego | 2 | 10/5/2014^29^ |
| San Cristóbal | 2 | 20/7/2014^29^ |
| Turrialba | 2 | 29/10/2014* |
| San Cristóbal | 2 | 5/3/2015^29^ |
| Turrialba | 2 | 8/3/2015* |
| Telica | 2 | 7/5/2015^32^ |
| Telica | 2 | 23/9/2015^32^ |
| Telica | 2 | 22/11/2015^32^ |
| Momotombo | 2 | 1/12/2015* |
| Masaya | 2 | 17/2/2016^29^ |
| San Cristóbal | 2 | 22/4/2016^29^ |
| Turrialba | 2 | 12/5/2016* |
| Poás | 3 | 12/4/2017^33^ |
| Fuego | 3 | 5/5/2017^29^ |
| Rincón de la Vieja | 2 | 23/5/2017* |
| Rincón de la Vieja | 2 | 11/6/2017* |
| San Cristóbal | 3 | 18/8/2017^29^ |
| Fuego | 4 | 3/6/2018^29^ |
| Rincón de la Vieja | 2 | 17/1/2019* |

* This study

References

1. Newhall, C. & Self, S. The Volcanic Explosivity Index (VEI): An Estimate of Explosive Magnitude for Historical Volcanism. *J. Geoph. Res.* **87**, 1231–1238 (1982).

2. Hill, D. et al. Seismicity remotely triggered by the magnitude 7.3 Landers, California, earthquake. *Science (80-. ).* **260**, 1617–1623 (1993).

3. Ye, L., Lay, T. & Kanamori, H. Large earthquakes rupture process variations on the Middle America megathrust. *Earth Planet. Sc. Lett.* **381**, 147–155 (2013).

4. Hill. D., Pollitz, F. & Newhall, C. Earthquake-volcano interactions. *Phys. Today* **55**, 41–47 (2002).

5. Manga, M. & Brodsky, E. Seismic Triggering of eruptions in the Far Field: Volcanoes and Geysers. *Annu. Rev. Earth Planet. Sci.* **34**, 263–291 (2006).

6. Fujita, E. et al. Stress field change around the Mount Fuji volcano magma system caused by the Tohoku megathrust earthquake, Japan. *Bull. Volc.* **75**, 679 (2013).

7. Ye, L., Lay, T., Kanamori, H. & Rivera, L. Rupture characteristics of major and great (Mw ≥ 7.0) megathrust earthquakes from 1990 to 2015: 1. Source parameter scaling relationships. *J. Geoph. Res.* **121**, 826–844 (2016).

8. Andrews, B. Magmatic storage conditions, decompression rate, and incipient caldera collapse of the 1902 eruption of Santa María volcano, Guatemala. *J. Volc. Geoth. Res.* **282**, 103–114 (2014).

9. Escobar-Wolf, R., Otoniel, M. and Rose, W. Notes on a New Geologic Map of Santiaguito Dome Complex, Guatemala. *Geol. Soc. Amer.* **8**, 2 (2010).

10. Roggensack, K. Unraveling the 1974 eruption of Fuego volcano (Guatemala) with small crystals and their young melt inclusions. *Geology* **29**, 911–914 (2001).

11. Lechner, H., et al. Magma storage and diking revealed by GPS and InSAR geodesy at Pacaya volcano, Guatemala. *Bull. Volc.* **81**, 18 (2019).

12. Scarlato, P. et al. The 2013 eruption of Chaparrastique volcano (El Salvador): Effects of magma storage, mixing, and decompression. *Chem. Geol.* **448**, 110–122 (2017).

13. Jiménez, D., et al. Spatio-temporal hazard estimation in San Miguel volcano, El Salvador. *J. Volc. Geoth. Res.* **358**, 171–183 (2018).

14. Robidoux, P., Aiuppa, A., Rotolo, S., Rizzo, A., Hauri, E. & Frezzotti, M. Volatile contents of mafic-to-intermediate magmas at San Cristóbal volcano in Nicaragua. *Lithos* **272**–**273**, 147–163 (2017).

15. Venugupal, S., Moune, S. and W.-J. Investigating the subsurface connection beneath Cerro Negro volcano and the El Hoyo Complex, Nicaragua. *J. Volc. Geoth. Res.* **325**, 211–224 (2016).

16. Diez, M., et al. Evidence for static stress changes triggering the 1999 eruption of Cerro Negro volcano, Nicaragua and regional aftershock sequences. *Geoph. Res. Lett.* **32**, L04309 (2005).

17. Obermann, Al., et al. Structure of Masaya and Momotombo volcano, Nicaragua, investigated with a temporary seismic network. *J. Volc. Geoth. Res.* **379**, 1–11 (2019).

18. Avellán, D. R. et al. Stratigraphy, chemistry, and eruptive dynamics of the 12.4 ka plinian eruption of Apoyeque volcano, Managua, Nicaragua. *Bull. Volc.* **76**, 792 (2014).

19. Mauri, G., et al. A geochemical and geophysical investigation of the hydrothermal complex of Masaya volcano, Nicaragua. *J. Volc. Geoth. Res.* **227**–**228**, 15–31 (2012).

20. Saballos, J., Conde, V., Malservisi, R., Connor, C., Alvarez, J. & Muñoz, A. Relatively short-term correlation among deformation, degassing, and seismicity: a case study from Concepción volcano, Nicaragua. *Bull. Volc.* **76**, (2014).

21. Borgia, A. and van Wyk de Vries, B. The volcano-tectonic evolution of Concepción, Nicaragua. *Bull. Volc.* **65**, 248–266 (2003).

22. Nuñez, E., et al. Crustal Velocity Anomalies in Costa Rica from Ambient Noise Tomography. *Pure Appl. Geoph.* **177**, 941–960 (2020).

23. Molina, F., et al. Stratigraphy and structure of the Cañas Dulces caldera (Costa Rica). *Geol. Soc. Amer.* **126 (11**–**12**, 1465–1480 (2014).

24. Villegas, R., Petrinovic, I. and Carniel, R. S-wave velocity zones at the Irazú Volcano (Costa Rica). *J. South. Amer. Earth Sci.* **90**, 314–324 (2019).

25. Namiki, A., Rivalta, E., Woith, H. & Walter, T. Sloshing of a bubbly magma reservoir as a mechanism of triggered eruptions. *J. Volc. Geoth. Res.* **320**, 156–171 (2016).

26. Namiki, A., Rivalta, E., Woith, H. Willey, T., Parolai, S & Walter, T. Volcanic activities triggered or inhibited by resonance of volcanic edifices to large earthquakes. *Geology* **47**, 67–70 (2018).

27. Paolucci, R. Amplification of earthquake ground motion by steep topographic irregularities. *Earth. Eng. Struc. Dyn.* **31**, 1831–1853 (2002).

28. Sulpizio, R. & Massaro, S. Influence of stress field changes on eruption initiation and dynamics: a review. *Front. Earth Sci.* **5**, 18 (2017).

29. Global Volcanism Program. in *Volcanoes of the World, v.4.6.6.* (ed. Venzke, E.) (Smithsonian Institution, Washington, DC, 2013). doi:10.5479/si.GVP.VOTW4-2013.

30. Scolamacchia, T., Pullinger, C., Caballero, L., Montalvo. F., B. L. & H. G. The 2005 eruption of Ilamatepec (Santa Ana) volcano, El Salvador. *J. Volc. Geoth. Res.* **189**, 291–318 (2010).

31. INSIVUMEH. Actividad del volcán Santiaguito años 2014-2018. *Intern. Rep.* **14** (2018).

32. Roman, D. C. et al. Mechanisms of unrest and eruption at persistently restless Volcanoes: Insights from the 2015 Eruption of Telica Volcano, Nicaragua. *Geochem. Geophys. Geosyst.* **20**, 4162–4183 (2019).

33. Mora-Amador, R., Rouwet, D., González, G., Vargas, P. & Ramírez, C. Volcanic Hazard Assessment of Poás (Costa Rica) Based on the 1834, 1910, 1953–1955 and 2017 Historical Eruptions. in *Poás volcano: The pulsing heart of Central America volcanic zone* (ed. Tassi, F., Vaselli, F & Mora-Amador, R.) (Springer, 2019). doi:10.1007/978-3-319-02156-0_11.
